# Research Progress of Chinese Herbal Medicine Intervention in Renal Interstitial Fibrosis

**DOI:** 10.3389/fphar.2022.900491

**Published:** 2022-06-13

**Authors:** Xiao-Yuan Liu, Xu-Bin Zhang, Ya-Feng Zhao, Kai Qu, Xiao-Yong Yu

**Affiliations:** ^1^ Department of Nephrology, Shaanxi Provincial Hospital of Traditional Chinese Medicine, Xi’an, China; ^2^ Department of Orthopaedic, Xi’an Hospital of Traditional Chinese Medicine, Xi’an, China

**Keywords:** chronic kidney disease, renal interstitial fibrosis, Chinese herbal medicine, mechanism, research progress

## Abstract

Chronic kidney diseases usually cause renal interstitial fibrosis, the prevention, delay, and treatment of which is a global research hotspot. However, no definite treatment options are available in modern medicine. Chinese herbal medicine has a long history, rich varieties, and accurate treatment effects. Hitherto, many Chinese herbal medicine studies have emerged to improve renal interstitial fibrosis. This paper reviews the mechanisms of renal interstitial fibrosis and recent studies on the disease intervention with Chinese herbal medicine through literature search, intend to reveal the importance of Chinese herbal medicine in renal interstitial fibrosis. The results show that Chinese herbal medicine can improve renal interstitial fibrosis, and the effects of Chinese herbal medicine on specific pathological mechanisms underlying renal interstitial fibrosis have been explored. Additionally, the limitations and advantages of Chinese herbal medicine in the treatment of renal interstitial fibrosis, possible research directions, and new targets of Chinese herbal medicine are discussed to provide a basis for studies of renal interstitial fibrosis.

## Introduction

Chronic kidney disease (CKD) is a global public health problem with low public awareness, high prevalence rate and medical cost, and poor prognosis (Mills et al., 2015; Hill et al., 2016). In 2017, 697.5 million patients had CKD, representing 9.1% of the global population. The number of cardiovascular deaths due to CKD in 2017 was 2.6 million, accounting for 4.6% of all deaths worldwide, making CKD the 12th leading cause of death worldwide (GBD Chronic Kidney Disease Collaboration, 2020). Regardless of the etiology of CKD, the final pathologic outcome is renal fibrosis (Zhou et al., 2016; Szeto, 2017). Renal fibrosis is a dynamic, multifactorial process involving complex and overlapping sequences of initiation, activation changes, subsequent execution, and eventual progression (Zhou et al., 2013). Upon persistent injury, inflammatory infiltration constitutes the first step of fibrosis, which appear as cell proliferation, fibroblast activation, and phenotypic transformation of renal tubular epithelial cells and endothelial cells. After activation, fibrotic signals mediate the process. Various cytokines are involved, resulting in hypoxia, renal tubular atrophy, scar formation, and eventually, renal failure. Renal fibrosis includes glomerulosclerosis and renal interstitial fibrosis (RIF). RIF is the pathological outcome of the vast majority of CKD. However, no targeted, clear, and effective prevention and control measures for RIF exist. Therefore, early prevention, delay, and reversal of RIF is a hot spot of current global medical research (Liu, 2011). Recent clinical research shows that modern medicine has succeeded in improving RIF to a certain extent; however, the clinical effect is still unsatisfactory. Moreover, accumulating evidence shows that traditional Chinese medicine, including extracts and compound preparations, is quite effective in preventing and treating RIF. For example, studies have shown that Astragalus can reduce TGF-β1 expression and Smad2/3 phosphorylation in mice with unilateral ureteral obstruction (UUO), thereby antagonizing the epithelial-mesenchymal transition (EMT) process and improving RIF (Shan et al., 2016). Hk-2 human proximal tubule epithelial cells were treated with angiotensin II (ANG-II) to induce EMT. After treatment with Fuzheng Huayu prescription, miR-21 expression and AKT phosphorylation were reduced and EMT was reversed (Wang Q. L. et al., 2020). This article reviews the research progress of Chinese herbal medicine intervention in RIF, in order to provide target ideas and references for clinical research.

## Pathological Mechanisms of RIF

**FIGURE 1:**
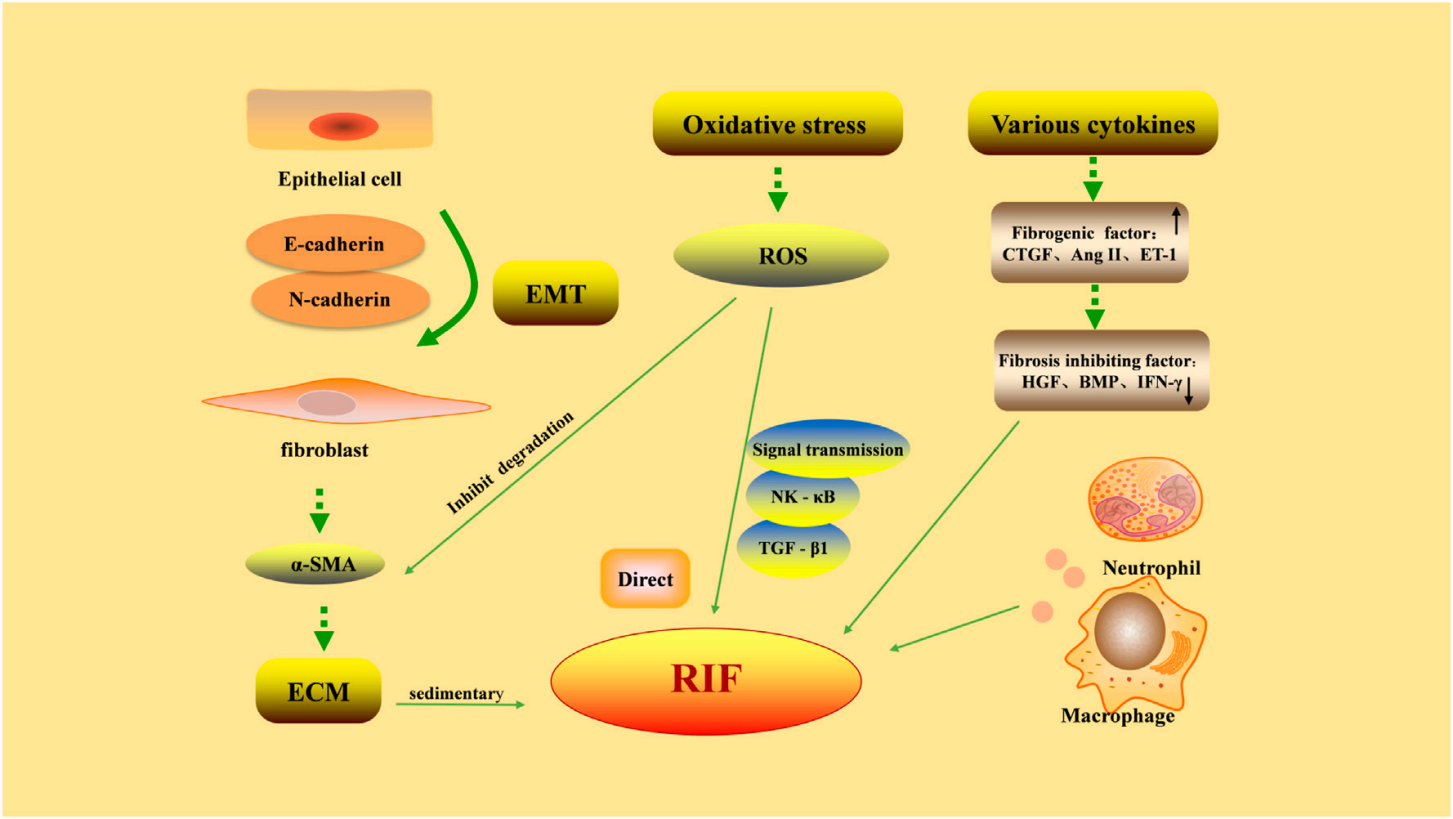
Pathological factors in renal interstitial fibrosis. Excessive deposition of extracellular matrix (ECM) is the main cause of renal interstitial fibrosis. Epithelial-mesenchymal transition (EMT) refers to the phenotypic transformation of epithelial cells to fibroblast-like cells by acquiring a mesenchymal morphology, through decreased expression of E-cadherin and increased expression of N-cadherin. The proliferation of fibroblasts is the precursor of ECM overproduction. The factors promoting/inhibiting fibrosis restrict each other, and the dynamic balance is lost, leading to the formation of fibrosis. The damaged part of renal interstitium can be rapidly infiltrated by a large number of inflammatory cells, aggravating fibrosis. The abnormal increase in reactive oxygen species (ROS) generation during oxidative stress can directly induce pathological damage to various renal cells and reduce the degradation of ECM by mesangial cells. On the other hand, ROS can participate in intracellular signaling pathways as a signaling molecule, causing RIF.

The pathological process of RIF is complex and related to the increase of extracellular matrix (ECM), EMT, oxidative stress, and the effect of various cytokines (Figure 1).

### Increased ECM

Similar to all other organs, the hallmark of renal fibrosis is the excessive sedimentation of ECM. ECM is a very intricate network structure consisting of collagen, elastin, a variety of glycoproteins, and other components, which constitute the basement membrane and interstitial space. In addition to providing scaffolding and organ stability, ECM has many other functions, including its role in phlogosis. The composition of ECM depends on the functions of the respective kidney chamber (ie., glomeruli, tubulointerstitium, and blood vessels). Clinically, ECM plays a significant role in CKD. It can participate in the occurrence of uncommon kidney diseases, promote renal fibrosis, and thus accelerate the pace of CKD. The catabolism of ECM is mainly associated with two substances, matrix metalloproteinases and tissue inhibitors of metalloproteinases, which are disordered when fibrosis occurs (Bulow and Boor, 2019). ECM plays a very important role in maintaining normal tissue structure and function as well as the process of cell growth and differentiation. It is in a dynamic balance of continuous metabolic renewal, degradation, and remodeling. Excessive deposition of ECM is the main cause of RIF (Razzaque and Taguchi, 2002). ECM overdeposition and sedimentation are significant characteristic landmarks of fibrosis (Djudjaj and Boor, 2019).

### Renal Tubular Epithelial Cell Phenotype Transformation

Renal tubular epithelial phenotypic transformation (EMT) is characterized by the transformation of the epithelial phenotype into a fibroblast-like mesenchymal phenotype, in which E-cadherin expression is reduced, and N-cadherin expression is increased. Epithelial cells lose top base polarity and, on the other hand, intercellular adhesion and gain mesenchymal properties (Liu et al., 2020; Li L. et al., 2021). This change is critical, not just in renal fibrosis, but in various biological processes such as cancer progression, organization palingenesis, embryo growth, and wound coalescence. In RIF, the effect of EMT is very clear: a large amount of evidence shows that EMT is a pivotal step in the launch of renal interstitial myofibroblasts. Thus, the precaution and treatment of EMT is a new direction in the study of renal fibrosis. Numerous studies have shown that EMT has three main effects on renal injury: affecting TEC role, leading to G2 stage cell period stasis, and dysregulating the balance between repair and fibrosis. As such, EMT is considered one of the most important processes leading to interstitial fibrosis (Liu, 2006; Thiery et al., 2009; Zhou et al., 2022).

### Oxidative Stress

Oxidative stress accelerates the progression of RIF, meaning that when the body is exposed to adverse stimuli, the balance between the oxidant and antioxidant systems in the body is lost, the generation of reactive oxygen species is abnormally increased, and the body is damaged by oxidative stress, causing damage in various ways. The increase of reactive oxygen species and the decrease of antioxidant enzyme activity are closely related to the production of obstructive renal injury. The accumulation of reactive oxygen species can directly cause pathological damage to various renal cells. In addition, it can stimulate the expression of fibrosis-related factors, accelerate the multiplication of fibroblasts, inhibit the degradation of ECM, and ultimately aggravate renal fibrosis (McCarty, 2006; Mittal et al., 2014; Ren et al., 2017). In addition, the excessive accumulation of reactive oxygen species will destroy the structure and function of cells, directly injuring the kidney. On the other hand, reactive oxygen species can be used as signaling molecules to participate in intracellular signaling pathways (such as nuclear transcription factor-κB [NF-κB]), and can influence key enzymes in kidney cells to initiate growth factor signaling and the transcription of a variety of cytokines, causing RIF (Rhyu et al., 2005).

### Renal Interstitial Fibroblast Proliferation, Activation, and Phenotypic Transformation

Renal interstitial fibroblasts are the main effector cells of fibrogenesis, and their massive proliferation and activation are the precursors for the production of excess ECM. Fibroblasts are reticulated in the kidney and are positioned between capillaries and epithelial cells to reinforce organization structure. Fibroblasts are star-shaped and contain a very dense endoplasmic reticulum, collagen particles, and many actin filaments. Interstitial fibroblasts are linked to the tubular and capillary basement membrane, which is facilitated by many cells. When inactive, stromal fibroblasts can generate erythropoietin. They can also secrete small proteins, such as fibroblast specific protein 1. Under normal conditions, fibroblasts produce a moderate amount of ECM to maintain stromal stability. However, under pathological conditions, fibroblasts can be activated by pro-fibrotic cytokines and certain stresses, generate α-SMA, causing an excessive ECM accumulation and obtaining myofibroblast phenotype. Myofibroblasts are the main cells that synthesize TGF-β1, and their number is closely related to the degree of RIF. They also secrete fibronectin, which provides scaffolds for the deposition of other ECM components and the formation of collagen fibers, leading to RIF (Eddy, 1996; Liu, 2011).

### Cytokines and Inflammatory Cells

Factors that promote fibrosis mainly include TGF-β1, endothelin-1 (ET-1), platelet-derived growth factor (PDGF), and tumor necrosis factor (TNF-α). Some of the main factors that inhibit fibrosis are hepatocyte growth factor (HGF), interferon-γ (IFN-γ), and bone morphogenetic protein (BMP). The promoting and inhibitory factors restrict each other and form a dynamic balance, thereby maintaining the normal morphological structure and function of the kidney. When the promoting effect is enhanced or the inhibitory effect is weakened, this dynamic balance is lost, resulting in the formation of fibrosis. A large number of inflammatory cells such as macrophages, lymphocytes, and monocytes can infiltrate rapidly into the damaged renal interstitium. Macrophages play an important role in the process of renal fibrosis, and many related studies have been conducted in recent years, showing that the extent of macrophage infiltration is positively correlated with renal disease. Following renal injury, macrophages change from M1 type to M2 type and express factors that promote fibroblast activation. On the other hand, macrophages can directly take part in the production of ECM, and excessive deposition of ECM aggravates RIF (Pan et al., 2015; Wang Y. Y. et al., 2017; Yan et al., 2021). TNF-α is also involved in RIF as a key player that mediates inflammatory reaction in multiple cells, such as macrophages, mesangial cells, and renal tubular epithelial cells. TNF-α can launch NF-κB, MAPK, and other signaling pathways, resulting in fibrosis. These signaling pathways further promote the activation of TNF-α by increasing the extent of many inflammatory factors. Serum TNF-α standard is closely related to the severity of renal injury (Liu Y. et al., 2021; Taguchi et al., 2021).

## Mechanisms of RIF Intervention

RIF refers to many signaling pathways and a variety of cytokines; the pathological process is complex and dynamic. There are many studies on the intervention of Chinese herbal medicines in RIF, including single herbs, Chinese herbal extracts, and Chinese herbal compound preparations. Specific mechanisms of intervention in RIF are summarized in this section.

### Regulation of TGF-β Signaling Pathway

In 1985, studies of the TGF-β family appeared, including molecular cloning of its signal transduction mediators. After this, researchers used cloning and genetic, biochemical, and other methods to identify similar polypeptides of TGF-β1, which constitute members of the TGF-β family. A total of 33 TGF-β -associated polypeptides are encoded in the mammalian genome based on the completed gene sequencing (Derynck et al., 1985). TGF-β belongs to the category of dimer peptides and has many functions, including the regulation of cell multiplication and cell differentiation and participating in the immune response. Numerous lines of evidence, including upregulation of TGF-β signaling in the glomerulus or tubulointerstitium, increased TGF-β-induced renal fibrosis, and amelioration of renal fibrosis through anti-TGF-β treatment, support the role of TGF-β in fibrosis associated with kidney diseases. TGF-β competence was significantly increased in glomerular diseases. TGF-β1 expression was also positively correlated with fibrosis in biopsy specimens (Yoshioka et al., 1993). TGF-β1 first binds to the membrane-bound TGF-β1 type II receptor and then activates the Smad signal. It is mainly responsible for the accumulation of ECM, not only by upregulating the gene of ECM but also by enhancing the production of plasminogen activator inhibitors and other substances, aggravating the production of ECM, and further inducing fibrosis (Li Z. et al., 2018). In addition, TGF-β1 can aggravate the progression of RIF through the induction of EMT (Yan et al., 2019). TGF-β can reduce the decomposition of the cell matrix and increase the composition of metalloproteinase inhibitors by decreasing the composition of metalloproteinases (Isaka, 2018). TGF-β itself is a very critical profibrotic factor, but its role can be affected during the fibrosis process. Cytokines such as IL-1 promote the effects of TGF-β. Conversely, certain substances, including vitamin D receptors, inhibit TGF-β activity and thus the activation of fibroblasts (Wu et al., 2009; Palumbo-Zerr et al., 2015).

In traditional Chinese medicine, comfrey has been widely used for thousands of years and is mainly produced in Xinjiang, China. Comfrey has a variety of pharmacological effects, including anti-inflammation, antiviral, and anti-tumor activities. Studies have reported that shikonin can significantly prevent the loss of E-cadherin in diabetic nephropathy (DN) mice, attenuate the expression of TGF-β1-induced mesenchymal markers, and inhibit TGF-β1/Smad-mediated EMT (Li Z. et al., 2018). Xiexin soup contains rhubarb, rhizoma coptidis, and Scutellaria baicalensis and has been used to treat diabetes for years. According to the pharmacodynamic evaluation, Coptis alkaloids (A), Rhubarb polysaccharides (P), and Scutellaria flavonoids (F) were identified as the main active ingredients, namely the APF components. APF negatively regulated the TGF-β1/Smad pathway in DN mice and decreased NF-κB phosphorylation in the kidney of mice, suggesting that APF inhibits NF-κB signaling and its effect on inflammation. APF is a combination of herbs that can achieve therapeutic effects through multiple pathways (Wu et al., 2015). Dendrobium mixture can reduce the level of glucose and lipids and improve insulin resistance. On the one hand, dendrobium mixture can lower blood glucose standard in DN DB/DB mice and, on the other hand, restrain TGF-β1/Smads passage, thereby inhibiting renal EMT and fibrosis (Chen et al., 2021a). Astragalus has a wide range of medicinal uses. Astragalus includes astragaloside IV, Astragalus polysaccharide, various amino acids, total astragalus saponins, carotenoids, and other components. Astragalus in UUO rats can partially prevent renal myofibroblast activation by preventing EMT in obstructive nephropathy, induce HGF expression, inhibit TGF-β1 expression, and significantly reduce renal interstitial fibrosis (Zuo et al., 2009). The traditional Chinese medicine compound preparation Huangqi Decoction improves renal interstitial fibrosis in a dose-dependent manner in UUO mice. The decoction can downregulate TGF-β/Smad conduction and recede the EMT process in addition to avoiding the excessive accumulation of ECM, thus postponing RIF (Zhao et al., 2016). Rhubarb and Astragalus Capsules, medicinal plants rhubarb and AM, attenuate apoptosis by regulating the p38 MAPK pathway and alleviate RIF in UUO rats (Zeng et al., 2020). Compound Jincao Granules is composed of Jincao, psyllium, corn silk, and Shiwei. It is a classic formula for the treatment of urinary calculi. This formula can prevent calcium oxalate crystal-induced kidney damage in mice by impacting the TGF-β/Smad conduction (Liu et al., 2020). Rhubarb is widely used in the treatment of CKD. Taking adenine-induced chronic tubulointerstitial fibrosis in rats as the research object, it was shown that rhubarb extract treatment could reduce renal damage and improve renal function. The rhubarb extract inhibited RIF mainly by regulating the TGF-β/Smad conduction (Zhang et al., 2018). TGF-β1 can act in an either autocrine or paracrine fashion. Safflower has a wide range of effects, including antioxidative and inflammation suppressive actions, and safflower extract can act by inhibiting autocrine TGF-β signaling, thereby inhibiting renal interstitial fibrosis in UUO rats (Yang et al., 2008). The traditional Chinese medicine compound Shenqi Jiedu granules is a commonly used formula for the treatment of CKD in the clinic. With astragalus, angelica, salvia, and other ingredients, it has the functions of invigorating the kidney, promoting blood circulation, and detoxifying. Research shows that, combined with P311, Shenqi Jiedu granules can reduce pathological changes such as RIF, tubular dilation, and atrophy in rat kidneys, and its mechanism may be related to EMT and the TGF-β-Smad-Ilk signaling pathway (Cai et al., 2017). From the perspective of traditional Chinese medicine theory, many scholars believe that “kidney Yang deficiency” is one of the reasons for the occurrence of renal fibrosis. “Tonifying kidney Yang deficiency” is also the main criterion in the prevention and treatment of RIF. Shenqi Pill is composed of Rehmannia glutinosa, Danpi, Poria, Chinese yam, Alisma, and other components. Shenqi Pill can improve renal interstitial fibrosis in rats with adenine-induced renal injury by regulating TGF-β1/Smads conduction (Chen et al., 2017). Another research showed that the traditional Chinese medicine prescription Yougui Pill could improve RIF by regulating TGF-β1/Smads conduction (Wang L. et al., 2015). Tanshinone IIA is the most abundant diterpene quinone in the rhizome of Salvia miltiorrhiza. It has been used for the treatment of CKD in many countries for more than 2000 years. Tanshinone IIA attenuated TGF-β/Smad and NF-κB conduction and inhibited inflammation, thereby reducing RIF in 5/6 nephrectomized rats with CKD (Wang D. T. et al., 2015). Saponins are a class of phytochemicals that exist in a variety of plants, including ginsenosides. Previous studies have suggested that total ginsenosides have a profound protective effect in acute myocardial ischemia, and ginsenosides can simultaneously inhibit TGF-β1/Smad, NF-κB conduction, activation of Nrf2-ARE conduction to attenuate renal fibrosis (Gao et al., 2021). Many patients with hypertension will develop CKD and experience the pathological changes of RIF, leading to renal failure. Qingxuan Antihypertensive soup is a Chinese herbal medicine formula that can significantly reduce patients’ hypertension. Previous research has reported that it can reduce the excessive accumulation of ECM and thereby reduce RIF by decreasing the TGF-β1/Smad conduction in spontaneously hypertensive rats (Liu W. et al., 2017). The Shenkang VII recipe is a commonly used composition for the treatment of CKD. Studies have shown that it can improve renal function by reducing ECM deposition in the kidney and the expression of inflammatory mediators. In addition, the Shenkang VII recipe inhibited the activation of TGF/Smad, NF-kB, and SHH signal transduction in UUO rats, slowing the progression of renal fibrosis (Zhou et al., 2020). Dioscorea, a common Chinese herbal medicine. β-hydroxybutyric acid (β-HB) (10 mM) to induce renal interstitial fibroblast (NRK) cells. Studies have shown that diosgenin inhibited TGF-β signaling pathway and antagonized EMT, thereby reducing RIF (Liu et al., 2012). Hirudin has a clear medicinal effect and is a commonly used thrombin inhibitor. Hirudin can improve RIF and has many advantages, including safety and low cost, which has attracted the attention of many researchers. Hirudin can inhibit inflammation and reduce the activation of the TGF-β pathway, thereby inhibiting EMT and improving RIF (Xie et al., 2020; Lin et al., 2022).

### Regulation of NF-κB Signaling Pathway

NF-κB protein has its own family and plays important roles in the body, including inflammatory response and immune processes. Transcription factors adjust the function of multiple genes including cell growth, development, and death (Vallabhapurapu and Karin, 2009). Oxidative stress can lead to RIF through the NF-κB pathway. When epithelial cells are hypoxic, the NF-κB pathway is activated. The main function of the NF-κB pathway in the body is to cause inflammation and promote fibrosis of tissues (Liu M. et al., 2017). The NF-κB pathway also regulates the activation of EMT-related Snail1 and exacerbates fibrosis. Transglutaminase promoter is an inducer that increases TGF-β expression and, on the other hand, accelerates ECM accumulation and exacerbates fibrosis by enhancing NF-κB expression through a positive feedback pathway (Bitzer et al., 2000; Chen et al., 2008; Ghiazza et al., 2010). Toll-like receptor 4 (TLR4) is an inflammatory stimulator and the acceptor of lipopolysaccharide (LPS), which is a significant element of the external membrane of Gram-negative bacteria that can irritate the activation of inflammatory conduction. Compared to other transcription factors, NF-κB is relatively conserved and plays an important role in many processes, including post-infection regulation of the immune system. The NF-κB signaling pathway can be activated by TLR4. There are a great many pathways that can initiate NF-κB signaling, which can be categorized as the canonical and noncanonical pathways. Among them, the activation of inflammatory receptors is a canonical pathway. Several TNF receptor members recruit TRAF2 and TRAF3 signals to initiate the NF-κB signal in a non-canonical fashion (Morgan and Liu, 2011). In mammals, the most widely studied member of the NF-κB/Rel family is P65, whose activation is influenced by multifarious stimuli and is associated with a variety of cells, such as inflammatory and apoptotic factors (Yde et al., 2011; Basak et al., 2012). In addition, activated p65 has been implicated in various inflammatory diseases, such as RIF (Fujihara et al., 2007). NF-κB is a hinge intermediary in inflammatory infiltration, which is mediated by excessive cell multiplication, ECM accumulation, and apoptosis (Barkett and Gilmore, 1999).

Curcumin is a chemical produced by the rhizome of turmeric with pharmacological effects of antioxidant, anti-fibrotic, anti-inflammatory, and anti-proliferative activities. The results of previous research showed that curcumin reduced RIF in UUO mice, mainly preventing the EMT process, inhibiting inflammatory response, and slowing down the excessive accumulation of ECM (Wang Z. et al., 2020). Fuzheng Huayu prescription is a commonly used Chinese herbal medicine prescription in the clinic. The role of the prescription is to promote blood circulation and remove blood stasis, replenish essence, and nourish yin. Studies have shown that it can attenuate mercuric chloride-induced renal interstitial fibrosis in rats by resisting oxidative stress and regulating the NF-κB signaling pathway (Yuan et al., 2017). Artemisinin is a very good antimalarial drug and is widely used around the world. Artemisia annua has many other functions, such as regulating the body’s immune function. Studies have shown that artemisinin can reduce RIF in rats with 5/6 nephrectomy by downregulating the NF-κB/NLRP3 signaling pathway, thereby providing renal protection (Wen et al., 2019). Zhenwu Decoction is a traditional Chinese medicine prescription with definite clinical efficacy. PPARγ has a variety of biological effects, including antagonizing the activation of TGF-β1 and NF-κB signals and the multiplication of mesangial cells. PPARγ also has biological functions such as anti-inflammatory and anti-fibrotic activities and regulation of lipid metabolism. Zhenwu Decoction inhibited the activation of the TGF-β1 signaling pathway and promoted the activity of PPARγ in UUO rats, thereby improving RIF (Li S. et al., 2018). Maslinic acid, a pentacyclic triterpene, using UUO mouse kidney and NRK49F cells treated with TGF-β, maslinic acid can disturb the MyD88, inhibit Smad4 nuclear activity, and improve the changes of renal fibrosis. In addition, hawthorn acid reduced NF-κB signaling and improved fibrosis (Sun et al., 2021). Coreopsis is an ethnic medicine. Local Uyghurs consume it as an herbal tea to treat high blood pressure and diarrhea. Coreopsis have anti-inflammatory, lipid-regulating, and blood sugar-regulating effects. In diabetic kidney cells, NF-κB expression is significantly increased, and NF-κB can reach the nucleus, induce inflammatory infiltration, and accelerate the progression of renal fibrosis. Studies have shown that in high glucose-induced rat glomerular mesangial cells, coreopsis ameliorates fibrosis through the TGF-β1/SMADS/AMPK/NF-κB axis (Yao et al., 2019). Resveratrol, a polyphenol with anticancer, anti-inflammatory, and antioxidant properties, can modulate Hsp70 expression in the kidneys of 5/6 nephrectomized uremic rats, and, at the same time, inhibit NF-κB expression, thereby exerting a renal protective effect (Feng et al., 2020). Cordyceps sinensis is an entomopathogenic fungus, which has been widely used in the clinic for centuries with definite curative effects in heart palpitations, epilepsy, and convulsions in children. N6-(2-hydroxyethyl) adenosine (HEA), derived from cicadas, is a compound that has pharmacological activities such as antagonizing inflammation. Studies have shown that HEA exerts advantageous effects on UUO-induced RIF in mice by regulating the NF-κB/TGF-β1/Smad axis, inhibiting inflammation, and activating renal fibroblasts (Zheng et al., 2018). The clinical studies of Astragalus membranaceus are extensive. Astragaloside IV is a very important physiological component of Astragalus membranaceus, which has multifarious activities such as vasodilation, prevention of endothelial dysfunction, improvement of myocardial cell energy metabolism, as well as anti-inflammatory and antioxidant activities. Studies have shown that Astragaloside IV reduces ECM accumulation and inflammatory cell infiltration in UUO-induced renal fibrosis and significantly attenuates inflammatory response. On the other hand, it inhibited lPS-induced inflammatory infiltration in epithelial cells, decreased NF-кB signaling both *in vivo* and *in vitro*, thereby delaying RIF (Zhou et al., 2017b). Quercetin is a natural compound widely found in Chinese herbs such as jujube and sophorae. Researchers have studied the effect of quercetin on kidney injury in UUO mice. The results showed that quercetin inhibited NF-κB signal transduction, regulated M1/M2 macrophage polarization, and improved RIF (Lu et al., 2018).

### Regulation of MAPK-Related Signaling Pathways

Mitogen-activated protein kinase (MAPK) is a widely conserved, versatile protein that takes part in many cellular activities. Many stimuli outside the cell activate MAPK, and its activation appears in the order of MAPK kinase kinase (MAPKKK), MAPKK kinase (MAPKK), and MAPK. There are many members of the MAPK family, and more than four are well-known, including extracellular signal-regulated kinase 1/2 (ERK1/2), C-Jun-amino terminal kinase (JNK), P38, and ERK5 (Nishimoto and Nishida, 2006). The P38 MAPK pathway carries out signal transduction in cells and plays an important role by participating in the inflammatory infiltration and producing fibrotic substances and profibrotic mediators. P38 has been implicated in ECM synthesis in the pathogenesis of fibrosis (Lee et al., 2019). JNK signaling promotes the manufacturing of inflammation and profibrotic molecules by tubular epithelial cells, as well as the dedifferentiation of tubular cells towards a mesenchymal phenotype. JNK signaling pathway and other signaling pathways crosstalk and participate in physiological and pathological activities in the body. JNK signaling pathway is most closely related to the TGF-β/SMAD signaling pathway. When activated, JNK enhances the activity of the TGF-β signaling pathway. Therefore, inhibition of p38 MAPK or TGF-β1 protein expression may be an effective strategy to alleviate RIF (Grynberg et al., 2017).

Puerarin (PR) from the puerarin plant has been comprehensively used in the clinic to treat a variety of diseases, including cardiovascular, brain, and lung injuries. A study showed that puerarin could inhibit the activation of MAPK in UUO mice and attenuate RIF (Zhou et al., 2017a). Gardeniside, an iridoid glycoside compound, is one of the most active components obtained from gardenia fruit. Autophagy is the process of recycling damaged cells and proteins and a very conserved cellular process that functions to maintain intercellular homeostasis. Autophagy is an important therapeutic target for DN; studies have shown that geniposide can increase MAPK activity in DN mice, enhance ULK1-mediated autophagy response, reduce AKT activity, thereby blocking oxidative stress, phlegmonosis, and renal fibrosis in diabetic kidneys (Dusabimana et al., 2021). Kangxianling is a traditional Chinese herbal formula that can cause more than 1,000 characteristic genes to be upregulated in azithromycin nephropathy rats, triggering the downstream launch of Wnt, TGF-β, and MAPK pathways to achieve the inhibition of RIF (Jiang et al., 2020). Astragaloside IV, as one of the most important pharmacological components of Astragalus membranaceus, has a wide range of pharmacological effects, including antagonizing inflammation, lowering blood pressure and hypoglycemia, and protecting the myocardium. Studies have shown that it inhibits TGF-β1-induced ERK1/2, p38 MAPK, phosphorylation of JNK, and IkBa, suggesting that astragaloside IV exerts anti-fibrotic effects through MAPK and NF-kB signaling pathways (Che et al., 2015). The main medicine in huangkui capsule is Huangkui, a traditional Chinese medicine. Huangkui capsule can obviously improve the renal fibrosis of diabetic nephropathy patients. Modulation of oxidative stress and p38MAPK/Akt pathway reduces renal fibrosis in rats with diabetic nephropathy compared with lipoic acid (Mao et al., 2015). In ginkgo biloba injection, the main drug is ginkgo biloba, clinical application of which is mature and has proven effects in the treatment of cardiovascular diseases. Clinical studies on ginkgo biloba leaf are also extensive. Ginkgo biloba leaf can inhibit inflammation and apoptosis, thus preventing testicular damage. On the other hand, ginkgo biloba leaf can downregulate the p38 MAPK signaling pathway and antagonize organ fibrosis (Li et al., 2017; Wang R. et al., 2017; Gevrek et al., 2018; Wang A. et al., 2018). Recent studies have shown that Ginkgo biloba can effectively improve cisplatin-induced post-renal interstitial fibrosis in rats with AKI by inhibiting renal cell apoptosis, which is mediated by downregulating the p38MAPK/TGF-β1 and p38MAPK/HIF-1α signaling axes (Liang et al., 2021). Salidroside (Sal) is the main pharmacological component of rhodiola rosea in Chinese herbal medicine. Salidroside has many beneficial functions for the body, including antagonizing inflammation and protecting kidney. In addition, salidroside decreased the accumulation of ECM and inhibited the activity of TLR4/NF-κB and MAPK signaling pathways in UUO mice and HK-2 cells, delaying renal fibrosis (Li et al., 2019). The ERK signaling pathway can be activated by a variety of stimuli, including high glucose, and this activation can cause EMT. MicroRNAs (miRNAs) are non-coding, relatively short RNAs that have a role in adjusting cell function. Existing studies have shown that multifarious miRNAs are highly correlated with EMT. Mulberry leaf is a commonly used traditional Chinese medicine and is rich in ingredients. Pharmacodynamic studies have shown that mulberry leaf and its functional compounds have the potential to prevent the development of DN, and network pharmacology has confirmed that mulberry leaf has anti-diabetic activities. Further studies showed that mulberry leaf extract could reduce the pathological changes of EMT induced by high glucose through the inhibition of the NADPH oxidase/ROS/ERK signaling axis. In addition, in HK-2 cells, mulberry leaf could increase the expression of MiR-302a and inhibit ZEB1, thereby inhibiting EMT (Ji et al., 2019). Interleukin-11 (IL-11) has its own biological function, and it belongs to the IL-6 system. When TGF-β signal is activated, it can greatly promote the activation of IL-11 and other fibrosis-related genes. IL-11 causes ERK1/2 activation in organ fibrosis, but not the JAK/STAT pathway. After IL-11 induced ERK1/2 activation, mRNA translation and fibrosis protein expression were further promoted. This is inconsistent with TGF-β activation. Another study confirmed that osthole directly affects IL-11-induced ERK1/2 signaling and alleviates renal fibrosis (Wu et al., 2021). Poricoic acid is the main chemical component of poria cocos. In a recent study, NRK-49F cells induced by TGF-β1 were used as the research object. The results showed that Poricoic acid could inhibit the activation of PDGF-C, Smad3, and MAPK pathways, thus reducing the excessive accumulation of ECM and improving RIF (Li Q. et al., 2021).

### Regulation of Wnt/β-Catenin Signaling Pathway

The Wnt/β-catenin signaling pathway is important for many biological functions, such as promoting tissue production, maintaining cell stability, and the development of some diseases (MacDonald et al., 2009). Wnt/β-catenin signaling pathway also plays a significant role in renal disease, and many studies have reported that this signaling pathway is involved in the progression of diabetic nephropathy, adriamycin nephropathy, focal glomerulosclerosis and other diseases (Surendran et al., 2005; Dai et al., 2009; He et al., 2012; Naves et al., 2012). Different levels of Wnt/β-catenin and the interaction between Wnt/β-catenin signaling pathway and other pathways are important links leading to EMT. It is well established that the EMT process can directly lead to RIF. In conclusion, inhibition of Wnt/β-catenin signaling activity or blocking of this signal transduction is beneficial to alleviate RIF (Liu, 2010; He et al., 2013; Li et al., 2013). On the other hand, After activation of Wnt signal, β-catenin is activated accordingly, promoting the process of renal fibrosis. Wnt/β-catenin also regulates the activity of a variety of downstream mediators in cells, including snail 1, fibroblasts and macrophages 6 and components of the renin-angiotensin system, and others. When Wnt/β-catenin activation promotes RIF, it is not a single effect, but often combined with other signal transduction (Li S. S. et al., 2021).

Accumulating evidence suggests that Astragaloside IV (AS-IV) exerts renoprotective effects by anti-inflammatory action, reducing oxidative stress, and blocking NF-κB transmission, thereby inhibiting inflammatory infiltration, attenuating podocyte damage by modulating the MAPK pathway, and attenuating ROS produced to improve podocyte apoptosis. In addition, the study demonstrated that AS-IV can inhibit Wnt/β-catenin signal transduction in UUO rats, suggesting that astragaloside IV can reduce RIF and protect renal function (Wang et al., 2014). Qishen Yiqi Dropping Pill (QSYQ) has a good clinical effect on kidney disease, including astragalus, salvia, red sandalwood, and other drugs. β-catenin (β-catenin) is a key protein in Wnt signaling. Recent research indicates that QSYQ can reduce RIF in UUO rats due to the selective inhibition of β-catenin upregulation and downstream fibrotic effects (Zhou et al., 2016). Tangshenning is a compound preparation that can relieve the symptoms of edema and dysuria in DN patients. AS-IV is the main active ingredient of Tangshenning, which can prevent podocyte EMT in DN. Scholars have studied its effects on podocyte EMT and Wnt/β-catenin pathway. It inhibited the launch of the Wnt/β-catenin pathway in DN mice (Cui et al., 2021). Most Chinese medicine scholars believe that DN also includes blood stasis blocking collaterals and kidney collaterals. Studies have found significant ECM deposition in the tubulointerstitium and some glomeruli of DN rats, and high glucose could stimulate and induce the activation of Wnt/β-catenin pathway, leading to RIF in DN rats. Huayu tongluo can significantly inhibit the deposition of ECM and block the overresponse of the Wnt/β-catenin pathway, thus alleviating RIF (Bai L. et al., 2017). Curcumin can inhibit Wnt/β-catenin signaling in diabetic rats and attenuate the reaction of superoxide, TGF-β1, and fibronectin activity in renal mesangial cells by high glucose and alleviate the accumulation of ECM in diabetic nephropathy (Ho et al., 2016). Poria is a commonly used traditional Chinese medicine in clinical practice, and it has a series of biological effects, including inhibition of inflammation, regulation of blood lipids, and inhibition of oxidation. Using HK-2 cells and UUO mice as the research objects, the study showed that novel tetracyclic triterpenoids, namely pachylic acid ZC (PZC), pachylic acid ZD (PZD), and pachylic acid ZE (PZE), could block the overexpression of Wnt/β-catenin signal, thereby intercepting Smad3 phosphorylation and significantly attenuating RIF. Furthermore, PZC and PZD have stronger renoprotective effects compared to PZE (Wang M. et al., 2018). Salidroside is the main pharmacological component of rhodiola rosea, which has a variety of pharmacological effects, such as treating diabetes, inhibiting oxidative stress, and delaying aging. Using a mouse model of azithromycin nephropathy, the study showed that salidroside also has many pharmacological effects on the kidney. It can attenuate Wnt/β-catenin signaling, thereby reducing proteinuria, protecting podocyte, and protecting renal function. The results of this study demonstrated the renal protective effect of salidroside and laid the foundation for further studies on salidroside and kidney (Huang et al., 2019). Alisma is a well-known natural product with lipid-lowering and kidney-protecting properties. Triterpenoids are the main active ingredients. 25-O-methyl alismatil F (MAF) is a pharmacological component extracted from Alismatil alismatil. Scholars have studied the effect of MAF on normal mouse renal tubular epithelial cells (NRK-52E) induced by TGF-β1 and Angiotensin II (ANG) and normal mouse fibroblast (NRK-49F) EMT, confirming that it can selectively inhibit TGF-mediated Smad3 phosphorylation, enhance Smad7 expression, inhibit Wnt/catenin signaling pathway, thereby attenuating EMT and relieving renal interstitial fibrosis (Chen et al., 2018). Tripterygium wilfordii is a common Chinese medicine. Studies have shown that tripterygium wilfordii treatment can reduce the expression of WNT-1 and β-catenin in renal tissues in diabetic rats, thereby alleviating fibrosis (Chang et al., 2018).

### Regulation of PI3K/Akt/mTOR and JAK2/STAT3 Signaling Pathways

mTOR is a serine/threonine protein kinase, which plays an important role in regulating many cellular processes, mainly cell growth. mTOR forms two main complexes, mTOR compound 1 (mTORC1) and compound 2. In the body, mTORC1 is more important for the regulation of cell growth. MTORC1 relies on the phosphorylation of several downstream factors, including ribosomal protein S6 kinase β -1 (p70S6K) and other substances, for its physiological activity. mTORC1/p70S6K signaling has been reported to mediate EMT during DN. Astragalus invigorates qi and transports spleen. It has a wide range of clinical indications and safe and clear efficacy in certain conditions such as heart-related diseases, leucopenia, and diabetic nephropathy. Studies have shown the effects of astragaloside IV on EMT, involving the reduction of high glucose-induced EMT in renal tubular cells through mTORC1/p70S6K signaling and subsequent downregulation of transcription in HK-2 cells (Chen et al., 2019). The Janus kinase/signal transduction and transcription activator (JAK/STAT) pathway has many physiological functions, involving many growth factors and cytokines. The JAK/STAT pathway is involved in cell proliferation. JAK can activate STAT3 in response to TGF-β and other related cytokines, playing a role in promoting fibrosis. Shenkang (SK) is a very common prescription for the treatment of renal failure. In UUO mice, studies have shown that SK can inhibit the conduction of JAK2/STAT3 signaling and significantly improve RIF in mice (Qin et al., 2021). Some studies have shown that autophagy promotes the occurrence of diabetic nephropathy and plays an important role in the progression of diabetic nephropathy. At present, there are many researches on autophagy, which can keep cells stable by clearing damaged cells and proteins from the body. Adjustment of autophagy within the body involves many signal pathways and targets; the most classic pathway is the PI3K/AKT/mTOR axis, and the most common target is mTOR. mTOR can adjust autophagy in two opposite directions. mTOR is downstream of the PI3K/AKT axis, which determines the activity of mTOR. The mixture of Dendrobium can regulate the PI3K/AKT/mTOR signal conduction to interfere with autophagy, inhibit kidney fibrosis, delay DN progression, and protect the renal function (Chen et al., 2021b).

### Regulation of Hedgehog and Notch Signaling Pathways

In mammals, the hedgehog signaling pathway is also very important in a variety of cellular signaling processes, particularly in embryonic cells. This pathway plays a pivotal role in the growth and development of animals. Shh is one of the important protein ligands of hedgehog proteins. Previous research has indicated that Shh activity increased significantly during fibrosis, suggesting a potential relationship between organ fibrosis and abnormal Shh signaling. Studies have shown that blocking the activation of Shh signaling helps inhibit RIF and prevent CKD progression. Polygonum cuspidatumt inhibits inflammatory infiltration and antagonizes oxidative stress, inhibiting tumor progression. Polysaccharides are an important pharmacological component of Polygonum cuspidatumt. Using UUO mice, researchers have studied the medicinal value of polysaccharides (BPPs) in Polygonum cuspidatum for relieving RIF and found that, BPP treatment could reduce ECM components and the activation of fibroblasts that produce these ECM components, resulting in a significant reduction in interstitial fibrosis. After BPP administration, the level of matrix metalloproteinase enzymes increased significantly in the body. In contrast, the levels of tissue inhibitors of metalloprotease were significantly reduced. The same case applied in HK-2 cells treated with TGF-β1. Furthermore, BPP administration decreases the expression of multiple transcription factors that regulate E-cadherin expression. The activation of the hedgehog pathway, the degree of EMT, and the degree of fibrosis are positively correlated. Studies have shown that BPP administration inhibited the hedgehog signaling pathway. Therefore, BPPs can suppress the EMT process by attenuating the activity of the hedgehog signal conduction, thereby improving RIF (Briscoe, 2009; Jenkins, 2009; Ding et al., 2012; Rauhauser et al., 2015; Li L. et al., 2021). Sedum is extracted from rhodiola rosea and widely used in the clinic. Studies have shown that Sedum extract can inhibit hedgehog signaling pathway and myofibroblast phenotypic transformation in UUO rats, thereby improving renal fibrosis (Bai Y. et al., 2017).

Many studies have shown that the Notch pathway can induce the EMT process and lead to fibrosis. Snail directly inhibits the transcription of E-cadherin, which brings about the loss of epithelial cell attachment and promotes the occurrence of EMT. Snail can regulate gene expression and is affected by a variety of signaling pathways, of which Notch signaling pathway is the most influential. Berberine (BBR) from Coptis chinensis and Phellodendron chinensis possesses a variety of pharmacological activities, including antibacterial, hypoglycemic, cholesterol-lowering, antitumor, and immunomodulatory properties. Using KKAy mice as animals to establish a DN mouse model, a study found that BBR administration may have indirec and direc pleiotropic effects on the Notch/Snail axis, inhibiting EMT, reducing RIF, and delaying the course of DN (Paznekas et al., 1999; Peinado et al., 2004; Katoh and Katoh, 2005; Herranz et al., 2008; Yang et al., 2017).

### Inhibition of NLRP3 Inflammasome and EZH2 Activity Expression

Inflammatory status can have an adverse effect on kidney diseases and aggravate RIF. Inflammatory corpuscle is a protein complex that acts as a receptor and regulates inflammatory factors in the body. The NOD-like receptor family has many members, including pyrin domain 3 (NLRP3), which is a typical inflammasome thar forms a complex composed of adaptor proteins, including the caspase recruitment domain (ASC) and the serine protease caspases, Apoptosis-associated speck-like protein of enzyme 1 (Casp1). Renal tubular epithelial cell injury is caused by many factors, such as insufficient perfusion and severe obstruction; these injuries activate the NLRP3 inflammasome, which regulates the production of proinflammatory cytokines. The NLRP3 inflammasome has its own characteristics; it can respond to various non-exogenous harmful signals, which makes this inflammasome widely explored in the study of renal diseases. Danggui Buxue decoction was founded in the Jin Dynasty of China. The main components are Angelica sinensis and Astragalus root in a ratio of 1:5, which shows significant renal protection. Danggui Buxue Decoction can reduce RIF in UUO rats. The reason for this beneficial effect is that it affects the activity of NLRP3 and inhibits inflammatory infiltration (Wang et al., 2016). *In vitro* test results showed that andrographolide effectively inhibited high glucose-induced apoptosis and EMT. In addition, it can reduce HK-2 cell death. The reason is that andrographolide can inhibit the activation of the NOD-like receptor family and NLRP3 inflammasome, antagonize the EMT process, and improve mitochondrial dysfunction. *In vivo*, andrographolide also plays a role in inhibiting EMT and improving RIF (Liu W. et al., 2021).

EZH2 regulates gene expression through epigenetics, participates in many biological processes, and catalyzes the trimethylation of lysine 27 of histone H3 (H3K27me3). The expression of EZH2 and H3K27me3 were elevated in obstructed kidneys, and inhibition of EZH2 attenuated RIF. Overexpression of EZH2 is associated with multiple cancerous tissue types (Simon and Lange, 2008; Yang and Yu, 2013). Reducing the expression of EZH2 is beneficial to reduce cell proliferation, antagonize EMT, and prevent tumor progression (Lu et al., 2010). Reducing the expression of EZH2 can weaken TGF-β1 activity (Xiao et al., 2016). Emodin, the main component of the traditional Chinese medicine rhubarb, can delay the progression of CKD. Researchers have studied the effect of emodin on tubulointerstitial fibrosis and its mechanism, and the results showed that emodin inhibits RIF in UUO rats, which was associated with the reduction of EZH2 activity *in vitro* and *in vivo* (Xu et al., 2021).

## Discussion

RIF is the final pathological outcome of almost all CKDs and is a determinant and prognostic indicator of CKD progression. Pathophysiologically, RIF includes several stages. Cell activation and injury are the first steps. The second stage includes fibrotic signaling, in which various cells factors and signal pathways that can crosstalk with each other are included. Next, it enters the fibrosis stage, and ECM accumulates pathologically. The last stage is the occurrence of renal damage (Eddy, 2000). Overcoming RIF has been a global challenge. Based on the molecular mechanisms and targets of RIF, studies of modern medicine has produced abundant clinical results, associating the classical signaling pathways such as TGF-β, macrophages, stem cells, and autophagy, and significant progress has been made. However, there are no treatment modalities that can completely prevent and treat RIF.

Traditional Chinese medicine has a long history and definite curative effect, which emphasizes the unity of nature and man. Therefore, researches on the treatment of RIF with Chinese herbal medicine are abundant. The intervention forms include original Chinese herbal medicines, Chinese medicine extracts, and Chinese medicine compound preparations, involving the types of drugs such as invigorating qi and nourishing yin, removing blood stasis, promoting blood circulation, clearing heat and removing dampness, invigorating the kidney and detoxifying, and supporting yang qi. Several Chinese herbal medicines can improve RIF in various ways, including astragalus and rhubarb. Chinese herbal medicine has great potential in the treatment of RIF. In terms of specific mechanisms, there are many studies on the regulation of various signaling pathways in RIF by Chinese herbal medicine, including the TGF-β/Smad, NF-κB, MAKP, Wnt/β-catenin, PI3K/Akt/mTOR, JAK2/STAT3, Hedgehog, and Notch signaling pathways. The TGF-β signaling pathway is the central signaling pathway in RIF. Downregulation of the TGF-β/Smad signaling pathway can inhibit EMT and accumulate ECM, thereby delaying the progression of RIF. Compared with Chinese herbal medicine, there are more clinical intervention studies on the TGF-β/Smad signaling pathway. However, it has also been shown that sustained inhibition of TGF- β has dual effects. Prolonged inhibition of the pathway may have adverse effects on the human body, such as affecting wound healing and antagonizing inflammation (Luangmonkong et al., 2017). Therefore, for TGF-β/Smad inhibitory intervention, it is advocated to be moderate, so as not to damage the normal physiological functions of the body. Similarly, for the regulation of other signaling pathways and cellular molecules, there may also be scales to achieve the purpose of improving RIF without affecting the body’s normal functions, but further research is needed to confirm this idea. In addition, Chinese herbal medicine has rich research on the intervention of the NF-κB, MAKP, and Wnt/β-catenin signaling pathways, which reflects the criticality and maturity of the above pathways in RIF research. On the other hand, Chinese herbal medicine can improve RIF by inhibiting the NLRP3 inflammasome and EZH2 gene expression. It can be seen that there are abundant studies on Chinese herbal medicine to delay RIF, and the overall efficacy is clear, as summarized in Tables 1–3. There are thousands of Chinese herbal medicines, with much more to be discovered and investigated. According to the existing research, Chinese herbal medicines, especially Chinese medicine compound preparations, have the advantages of multi-target intervention to improve RIF, and have broad research prospects. The advantages are worth further exploration. Current research has laid a solid foundation for the basic and clinical research of RIF in the future.

**TABLE 1:** Summary of Chinese herbal medicines that improve RIF-related mechanisms Single herb.

Chinese herbal medicine	Specific drug	Research object	Specific mechanism	References
Astragalus	Astragalus	UUO rat	↓TGF-β1/Smad	Zuo et al. (2009)
			↓ EMT	
			↑ HGF	
Rhubarb	Rhubarb	Chronic-renal tubulointerstitial fibrosis in rat	↓ TGF-β/Smad	Zhang et al. (2018)
Safflower	Safflower	UUO rat	↓ TGF-β	Yang et al. (2008)
Yam	Yam	β-Hydroxybutyric acid-induced fibroblasts	↓ TGF-β/Smad	Liu et al. (2012)
			↓ EMT	
Coreopsis	Coreopsis	Rat glomerular mesangial cell	↓TGF-β1/SMADS	Yao et al. (2019)
			↓AMPK/NF-κB	
Huangkui Capsules	Huangkui	DN rat	↓ p38MAPK/Akt	Mao et al. (2015)
Ginkgo biloba injection	Ginkgo biloba	Cisplatin- induced rat AKI	↓p38MAPK/TGF-1	Liang et al. (2021)
			↓p38MAPK/HIF1α	
Mulberry leaves	Mulberry leaves	HK-2	↓NADPH oxidase/ROS/ERK	Ji et al. (2019)
Astragalus	Astragalus	UUO rat	↓ TGF-β1	Shan et al. (2016)
			↓ EMT	
Tripterygium wilfordii	Tripterygium wilfordii	DN rat	↓Wnt-1/β-catenin	Chang et al. (2018)

↑: increase or activation; ↓: decrease or inhibition. UUO: unilateral ureteral obstruction; TGF-β1/Smad: transforming growth factor-beta1/Smad; EMT: Epithelial-mesenchymal transition; HGF: hepatocyte growth factor; AMPK: AMP-activated kinase protein; NF-κB: nuclear factor kappa beta; DN: diabetic nephropathy; p38MAPK: p38 mitogen-activated protein kinase; Akt: serine-threonine kinase; AKI: acute kidney injury; HIF-1α: hypoxia-inducible factor-1α; HK-2: human renal tubular epithelial cells; NADPH: nicotinamide adenine dinucleotide phosphate; ROS: reactive oxygen species; ERK: extracellular regulated protein kinases.

**TABLE 2:** Summary of Chinese herbal medicines that improve RIF-related mechanisms Chinese herbal extract.

Chinese herbal medicine	Specific drug	Research object	Specific mechanism	References
Comfrey	Acetylshikonin	DN mouse	↑ E-cadherin	Li et al. (2018b)
			↓ TGF-β1/Smad	
			↓ EMT	
Salvia	Tanshinone	Nephrectomy in CKD rat	↓ TGF-β/Smad	Wang et al. (2015a)
			↓ NF-κB	
Ginseng	Ginsenosides	Natural aging rat	↓ TGF-β1/Smad	Gao et al. (2021)
			↓ NF-κB	
			↑ Nrf2-ARE	
Leech	Hirudin	UUO mice	↓ TGF-β	Lin et al. (2022)
			↓ EMT	
			↓ MCP-1	
Turmeric	Curcumin	UUO mice	↓ TLR4/NF-κB	Wang et al. (2020b)
			↓ PI3K/AKT	
Artemisia annua	Artemisinin	rats with 5/6 nephrectomy	↓ NF-κB/NLRP3	Wen et al. (2019)
Hawthorn	Hawthorn acid	UUO mice	↓ TGF-β	Sun et al. (2021)
			↓ NF-κB	
White hellebore	Resveratrol	5/6 nephrectomy uremic rat	↑ Hsp70	Feng et al. (2020)
			↓ NF-κB	
Cicada Cordyceps	N6-(2-hydroxyethyl)adenosine	UUO induced mice	↓ NF-κB	Zheng et al. (2018)
			↓ TGF-β1/Smad	
Astragalus	Astragaloside IV	UUO mice	↓ TLR4	Zhou et al. (2017b)
			↓ NF-κB	
Kudzu root	Puerarin	UUO mice	↓ MAPK	Zhou et al. (2017a)
Gardenia	Geniposide	DN mice	↑ MAPK	Dusabimana et al. (2021)
			↓ AKT	
Astragalus	Astragaloside IV	TGF-β1 induced mice	↓ MAPK	Che et al. (2015)
			↓ NF-κB	
Rhodiola	Salidroside	UUO mice	↓ TLR4/NF-κB	Li et al. (2019)
			↓ MAPK	
Cnidium	Osthole	UUO mice	↓ TGF-β/Smad2/3	Wu et al. (2021)
		HK-2 cells	↓ IL-11/ERK1/2	
Astragalus	Astragaloside IV	UUO rat	↓ Wnt/β-catenin	Wang et al. (2014)
Turmeric	Curcumin	DN rat	↓ Wnt/β-catenin	Ho et al. (2016)
Poria	Poria acid	HK-2 cells	↓ Wnt/β-catenin	Wang et al. (2018b)
		UUO mice		
Rhodiola	Salidroside	Azithromycin nephropathy mouse model	↓ Wnt/β-catenin	Huang et al. (2019)
Alisma	25-O-methylalisol F	AngiotensinII-induced normal mice	↓ Wnt/β-catenin	Chen et al. (2018)
Astragalus	Astragaloside IV	High-glucose-induced renal tubular cells	↓mTORC1/p70S6K	Chen et al. (2019)
Knotweed	Polysaccharide	UUO mice	↓ TGF-β1	Li et al. (2021a)
			↓ Hedgehog	
Coptis, Cork	Berberine	DN mice	↓ Notch/snail	Yang et al. (2017)
Andrographis	Andrographolide	Diabetic mice	↓ NLRP3	Liu et al. (2021a)
Rhubarb	Emodin	UUO rat	↓ EZH2	Xu et al. (2021)
Jujube	Quercetin	UUO mice	↓ NF-κB	Lu et al. (2018)
Rhodiola rosea	Sedum sarmentosum Bunge	UUO rat	↓Hedgehog	Bai et al. (2017b)
Poria cocos	Poricoic acid	NRK-49F	↓ PDGF-C	Li et al. (2021b)
			↓ TGF-β1	
			↓ Smad3	
			↓ MAPK	

↑: increase or activation; ↓: decrease or inhibition. Nrf2-ARE: nuclear factor erythroid 2-related factor 2-antioxidant response element; MCP-1: monocyte chemoattractant protein-1; TLR4: Toll-like receptor 4; PI3K: phosphatidylinositol 3 kinase; AKT: protein kinase B; NLRP3: NOD-like receptor protein 3; Hsp70: heat shock protein 70; MAPK: mitogen-activated protein kinase; IL-11: Interleukin-11; ERK1/2: extracellular regulated protein kinases; mTORC1: mammalian target of rapamycin complex 1; p70S6K: ribosomal protein S6 kinase β-1; EZH2: Enhancer of zeste homolog 2; NRK-49F: Rat normal kidney 49 fibroblast; PDGF-C: Platelet-derived growth factor C.

**TABLE 3:** Summary of Chinese herbal medicines that improve RIF-related mechanisms Compound and Compound extract.

Chinese herbal medicine	Specific drug (etc)	Research object	Specific mechanism	References
Dendrobium mixture	Dendrobium, Astragalus, Salvia	DNdb/db mice	↓ TGF-β1/Smad	Chen et al. (2021a)
			↓ EMT	
Astragalus Soup	Astragalus, Poria, Melon	UUO mice	↓ TGF-β/Smad	Zhao et al. (2016)
			↓ EMT	
Rhubarb Astragalus Capsules	Rhubarb, Astragalus	UUO rat	↓TGF-β1/p38	Zeng et al. (2020)
Compound MoneyGrass Granules	Moneygrass, plantain seeds, corn silk	Calcium oxalate crystal-induced kidney injury in mice	↓ TGF-β/Smad	Liu et al. (2020)
ShenqiJiedu Granules	Astragalus, Angelica, Salvia	UUO rat	↓ EMT	Cai et al. (2017)
			↓ TGF-Smad-Ilk	
Shenqi Pill	Rehmannia, Danpi, Poria	Adenine-induced renal injury in rat	↓ TGF-β1/Smads	Chen et al. (2017)
Uguimaru	Rehmanniaglutinosa, aconite, cinnamon	UUO rat	↓ TGF-β1/Smad	Wang et al. (2015b)
Qingxuan antihypertensive soup	BambooRu, Poria, Gentian Grass	Spontaneously Hypertensive Rat	↓ TGF-β1/Smad	Liu et al. (2017b)
			↓ ECM	
ShenkangⅦ recipe	Polyporus, Money Grass, Sea Sand	UUO rat	↓ TGF/Smad	Zhou et al. (2020)
			↓ NF-kB	
			↓ SHH	
FuzhengHuayu Recipe	Salvia, Schisandra, Peach Kernel	mercuric chloride induced rat	↓ NF-κB	Yuan et al. (2017)
Zhenwu Soup	Peony, Poria,Ginger	UUO rat	↓ TGF-β1	Li et al. (2018a)
			↑ PPARγ	
Anti - cellulite	Salvia, Jujube Achyranthes	Azithromycin nephropathy in rat	↓ Wnt	Jiang et al. (2020)
			↓ TGF-β	
			↓ MAPK	
QishenYiqi Dropping Pills	Astragalus, Salvia, Red Sandalwood	UUO rat	↓ β-catenin	Zhou et al. (2016)
removingblood stasisand dredging collaterals	Salvia, Dilong,Leech	DN rat	↓ Wnt/β-catenin	Bai et al. (2017a)
Shenkang	Safflower, Salvia, Astragalus	UUO mice	↓ JAK2/STAT3	Qin et al. (2021)
Dendrobium mixture	Dendrobium, Astragalus, Schisandra	DN rat	↓ PI3K/Akt/mTOR	Chen et al. (2021b)
Angelica sinensis soup	Astragalus, Angelica	UUO rat	↓ NLRP3	Wang et al. (2016)
FuzhengHuayu Soup	Cordycepssinensis powder, Danshen, Peach kernel	HK-2 cell	↓ miR-21	Wang et al. (2020a)
			↓ AKT	
			↓ EMT	
Xiexin soup	Coptisalkaloids, rhubarb polysaccharides	DN mice	↓ TGF-β1/Smad	Wu et al. (2015)	
Rhubarb, Coptis, Scutellaria	Scutellaria flavonoids		↓ NF- κB		
Tangshenning	AS-IV	DN mice	↓ Wnt/β-catenin	Cui et al. (2021)	
Astragalus, Rhubarb, Chuanxiong					

↑: increase or activation; ↓: decrease or inhibition. p38: p38 MAPK; TGF-Smad-Ilk: TGF-β1-Smad-ILK, pathway; ECM: extracellular matrix; SHH: sonic hedgehog signaling; PPARγ: Peroxisome Proliferator-Activated Receptor γ; JAK2/STAT3: Janus kinase2/signal transducer and activator of transcription3; mTOR: mammalian target of rapamycin; AS-IV: Astragaloside IV; miR-21:microRNA-21.

However, there are several limitations to the use of Chinese herbal medicines in RIF. For example, relatively few researches exist on the signaling pathways of RIF, such as Hedgehog and Notch, which need to be further elucidated. Second, a large number of molecules and mechanisms with pro-fibrotic or anti-fibrotic properties have been identified, and there are still many mechanisms in Chinese herbal medicine that have not been covered, which may also be a new direction for future research. Since fibrosis-related pathways or molecular mechanisms interfere with each other, finding targets that connect these factors may also be a new direction for Chinese herbal medicine intervention. In addition, most of the interventions of Chinese herbal medicine in RIF are reflected as the overall curative effect. It is necessary to deeply explore the pharmacological components and mechanisms of specific drugs, analyze the pharmacological effects and mutual effects of each drug in the compound preparation, and detect the optimal dose and administration regimens. This is an inevitable trend for Chinese herbal medicine to improve RIF, which has a positive impact on the therapeutic significance of Chinese herbal medicine and global acceptance.

## Concluding Remarks

To sum up, major breakthroughs and progress have been made in the research on Chinese herbal medicine to improve RIF, and the prevention and treatment of RIF is still a major challenge on a global scale. Because the RIF process is complex and involves dynamic changes, current research is limited in terms of the modeling time, and the inducing and influencing factors are single, the simulation research and research effect need to be further considered. Second, current research is based on cell and animal models; thus, much work is needed to elucidate clinical relevance. However, research thinking and methods continue to progress. Chinese herbal medicine has a long and profound history, and worthy of further exploration. Based on the research results of Chinese herbal medicine intervention in RIF, we believe that Chinese herbal medicine will greatly promote the progress in RIF prevention and treatment.
